# Structure of 3-ketoacyl-(acyl-carrier-protein) reductase from *Rickettsia prowazekii* at 2.25 Å resolution

**DOI:** 10.1107/S1744309111030673

**Published:** 2011-08-16

**Authors:** Sandhya Subramanian, Jan Abendroth, Isabelle Q. H. Phan, Christian Olsen, Bart L. Staker, A. Napuli, Wesley C. Van Voorhis, Robin Stacy, Peter J. Myler

**Affiliations:** a Seattle Structural Genomics Center for Infectious Disease (SSGCID), USA; b Seattle Biomedical Research Institute, 307 Westlake Avenue North, Seattle, WA 98125, USA; c Emerald BioStructures Inc., 7869 NE Day Road West, Bainbridge Island, WA 98110, USA; d Department of Biochemistry, University of Washington, Box 357742, Seattle, WA 98195, USA

**Keywords:** *Rickettsia prowazekii*, 3-oxoacyl-(acyl-carrier-protein) reductase, FabG, epidemic typhus, infectious diseases, SSGCID

## Abstract

*Rickettsia prowazekii*, a parasitic Gram-negative bacterium, is in the second-highest biodefense category of pathogens of the National Institute of Allergy and Infectious Diseases, but only a handful of structures have been deposited in the PDB for this bacterium; to date, all of these have been solved by the SSGCID. Owing to its small genome (about 800 protein-coding genes), it relies on the host for many basic biosynthetic processes, hindering the identification of potential antipathogenic drug targets. However, like many bacteria and plants, its metabolism does depend upon the type II fatty-acid synthesis (FAS) pathway for lipogenesis, whereas the predominant form of fatty-acid biosynthesis in humans is *via* the type I pathway. Here, the structure of the third enzyme in the FAS pathway, 3-­ketoacyl-(acyl-carrier-protein) reductase, is reported at a resolution of 2.25 Å. Its fold is highly similar to those of the existing structures from some well characterized pathogens, such as *Mycobacterium tuberculosis* and *Burkholderia pseudomallei*, but differs significantly from the analogous mammalian structure. Hence, drugs known to target the enzymes of pathogenic bacteria may serve as potential leads against *Rickettsia*, which is responsible for spotted fever and typhus and is found throughout the world.

## Abstract 2

The *R. prowazekii* 3-ketoacyl-(acyl-carrier-protein) reductase is similar to those from other prokaryotic pathogens but differs significantly from the mammalian orthologue, strengthening its case as a potential drug target.

## 1. Introduction

Epidemic typhus has afflicted humans for a long time, with the first written record going back to 1083 AD (Szybalski, 1999 ▶). The obligate bacterial parasite *Rickettsia prowazekii* is responsible for the disease, which is transmitted by various arthropod vectors, most commonly body lice (*Pediculus humanus humanus*) or flying squirrel fleas (*Orchopeas howardii*) and lice (*Neohematopinus sciuropteri*), through their saliva or feces. Epidemic typhus flourishes in areas with overcrowding or poor hygiene, such as jails or refugee camps. Although louse control has succeeded in suppressing typhus in the modern era, recent outbreaks in the USA (Reynolds *et al.*, 2003 ▶), Africa (Mokrani *et al.*, 2004 ▶) and Europe (Tarasevich *et al.*, 1998 ▶) have re-established *R. prowazekii* as a re-emerging disease threat; its potential use in bioterrorism (Radulovic & Azad, 2002 ▶) places it in category B of the Biodefense Category A, B and C Priority Pathogens of the National Institute of Allergy and Infectious Diseases.

The genome of *R. prowazekii*, a member of the Gram-negative alphaproteobacteria, is not large at just over 1.1 million base pairs, with only 834 open reading frames found (Andersson *et al.*, 1998 ▶). This reduced proteome is representative of the dependence of *Rickettsia* on its host for many biosynthetic functions and leaves fewer innate enzyme targets for antipathogenic targeting. One well conserved pathway that it shares with other bacterial pathogens is for fatty-acid biosynthesis as a primary source of cell-membrane lipids.

**Figure 1:**
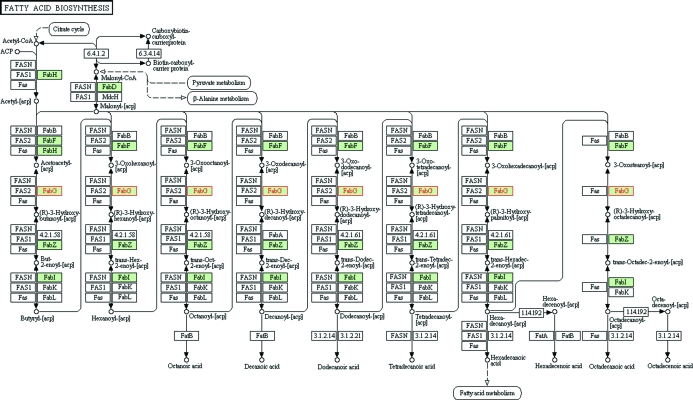
Fatty-acid biosynthetic pathway for *R. prowazekii*. The *R. prowazekii* fatty-acid biosynthesis map from the KEGG PATHWAY database (Kanehisa & Goto, 2000 ▶) is shown with FabG highlighted in red.

In *R. prowazekii* fatty-acid biosynthesis occurs *via* the type II fatty-acid synthase pathway, which is thought to be descendant from an algal plastid (Wilson, 2002 ▶) and consists of four to eight individual enzymes that start with acetyl coenzyme A and malonyl-(acyl-carrier-protein) to create elongated fatty acids for the lipid bilayer (Fig. 1 ▶). In contrast, human fatty-acid biosynthesis is performed by a single large multifunctional peptide, the multienzyme type I fatty-acid synthase, which passes the substrate from one domain to the next. 3-Ketoacyl-(acyl-carrier-protein) [3-ketoacyl-(ACP)] reductase, gene name *fabG*, is the third enzyme in the type II pathway and catalyzes the chemical reaction
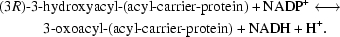
While many structures of 3-ketoacyl-(ACP) reductase have been solved, those from *Escherichia coli* (Price *et al.*, 2001 ▶), *Mycobacterium tuberculosis* (Cohen-Gonsaud *et al.*, 2002 ▶) and *Burkholderia pseudo­mallei* (PDB entry 3ftp; Seattle Structural Genomics Center for Infectious Disease, unpublished work) to name only a few, only five structures in total have been solved for *Rickettsia* and the solution of the structure of an essential pathway enzyme in this bacterial parasite holds promise for drug development against typhus.

## 2. Methods

### 2.1. Protein expression and purification

FabG from *R. prowazekii* strain Madrid E (NCBI NP_221114; *fabG* gene RP762; UniProt P50941; Pfam ID PF00106; EC 1.1.1.100) spanning the full-length protein from residues 1 to 241 was cloned from genomic DNA into a pAVA0421 vector using ligation-independent cloning (Aslanidis & de Jong, 1990 ▶) to produce a construct with an uncleaved N-terminal hexahistidine-affinity tag followed by the human rhinovirus 3C protease-cleavage sequence (sequence MAHHHHHHMGTLEAQTQGPGS-ORF). *R. prowazekii* FabG was expressed in *E. coli* using BL21(DE3)R3 Rosetta cells and autoinduction medium in a LEX bioreactor as follows. A 3 ml starter culture of LB broth with ampicillin was grown for ∼18 h at 310 K. ZYP-5052 auto-induction medium was freshly prepared as per Studier’s published protocols (Studier, 2005 ▶). Ampicillin was added to 2 l sterile auto-induction medium, which was inoculated with all of the overnight culture and then placed into the LEX bioreactor. The culture was grown for ∼24 h at 298 K; the temperature was reduced to 288 K and the culture was grown for a further ∼60 h. To harvest, the cultured medium was centrifuged at 4000*g* for 20 min at 277 K. The cell paste was flash-frozen in liquid nitrogen and stored at 193 K.

The frozen cells were resuspended in lysis buffer (25 m*M* HEPES pH 7.0, 500 m*M* NaCl, 5% glycerol, 30 m*M* imidazole, 0.025% sodium azide, 0.5% CHAPS, 10 m*M* MgCl_2_, 1 m*M* TCEP, 250 ng ml^−1^ AEBSF and 0.05 µg ml^−1^ lysozyme). The resuspended cell pellet was disrupted on ice for 30 min with a Virtis sonicator (408912; set at 100 W power with alternating cycles of 15 s pulse on and 15 s pulse off). The cell debris was incubated with 20 µl Benzonase nuclease (EMD Chemicals; 25 units ml^−1^) at room temperature for 45 min and clarified by centrifugation on a Sorvall SLA-1500 at 14 000 rev min^−1^ for 75 min at 277 K. The protein was purified from the clarified cell lysate by immobilized metal-affinity chromatography on an Ni–NTA HisTrap FF 5 ml column (GE Healthcare) equilibrated with binding buffer (25 m*M* HEPES pH 7.0, 0.5 *M* NaCl, 5% glycerol, 30 m*M* imidazole, 0.025% sodium azide, 1 m*M* TCEP) at 277 K. The recombinant protein was eluted with 250 m*M* imidazole. The protein was collected in the flowthrough and was further resolved by size-exclusion chromatography (SEC) using a HiLoad 26/60 Superdex 200 column (GE Healthcare) at 277 K. Pure fractions collected in SEC buffer (25 m*M* HEPES pH 7.0, 500 m*M* NaCl, 2 m*M* DTT, 0.025% sodium azide and 5% glycerol) as a single peak were pooled and the protein was concentrated to 25.35 mg ml^−1^, as determined *via* *A*_280_ including compensation for the absorption coefficient, with good solubility. The 2.5 ml sample was flash-frozen and stored at 193 K prior to crystallo­graphy.

### 2.2. Crystallization and X-ray data collection

For the FabG target at a protein concentration of 23.4 mg ml^−1^ in SEC buffer, two sparse-matrix screens were set up, JCSG+ (Emerald BioSystems) and PACT (Molecular Dimensions), following the  strategy of Newman *et al.* (2005 ▶). 0.4 µl protein solution was mixed with 0.4 µl well solution and equilibrated at 289 K against 100 µl reservoir solution using 96-well Compact Jr crystallization plates from Emerald BioSystems. Crystals suitable for diffraction studies were obtained within two weeks from JCSG+ screen condition D3: 100 m*M* sodium/potassium phosphate pH 6.2, 50% PEG 200, 200 m*M* NaCl. The crystals, which were approximately 0.1–0.15 mm in each direction, did not need additional cryoprotection and were flash-frozen by plunging them into liquid nitrogen.

A native data set was collected to 2.25 Å resolution in-house at Cu *K*α wavelength using a Rigaku SuperBright FR-E+ rotating-anode X-ray generator equipped with Osmic VariMax HF optics and a Saturn 944+ CCD detector (Table 1 ▶). Because of the rather long *c* axis (183 Å) and the rather small in-house detector (94.4 mm), the data set was collected with fine ϕ slicing (0.3°). The diffraction data were reduced with *XDS*/*XSCALE* (Kabsch, 2010 ▶) to 2.25 Å resolution. The crystals belonged to the primitive tetragonal space group *P*4_1_2_1_2. The data-collection and refinement statistics are summarized in Tables 1 ▶ and 2 ▶.

### 2.3. Structure solution and refinement

Using the *BLAST* (Altschul *et al.*, 1997 ▶) homology-search server at NCBI, *Brucella melitensis* glucose/ribitol dehydrogenase (PDB entry 3emk; Seattle Structural Genomics Center for Infectious Disease, unpublished work) was found to be the closest homolog with a known structure at the time, with 54% sequence identity. The monomer of PDB entry 3emk was modified with the *CCP*4 program *CHAINSAW* (Stein, 2008 ▶; Winn *et al.*, 2011 ▶), which deleted four residues (Glu77–Gly80) and mutated 107 residues to their last common atom. Based on a packing density *V*_M_ (Matthews, 1968 ▶) of 2.24 Å^3^ Da^−1^, corresponding to 45% solvent, two molecules were expected to be present in the asymmetric unit. Molecular replacement was performed with the *CCP*4 program *Phaser* (McCoy *et al.*, 2007 ▶) using the modified model of 3emk and data to 3 Å resolution. *Phaser* could position two molecules in the asymmetric unit with rotation/translation *Z* scores of 5.4/17.7 and 5.4/26.7, respectively, and an *R* factor of 0.52. The initial model was improved with *ARP*/*wARP* (Langer *et al.*, 2008 ▶), which built 367 residues in 13 chains with *R*_work_ = 0.259 and *R*_free_ = 0.307. This model was then extended and refined in iterative cycles of *REFMAC*5 (Murshudov *et al.*, 1997 ▶) and *Coot* (Emsley *et al.*, 2010 ▶). In *REFMAC*5 no data-cutoff criteria were used and NCS restraints were applied given the limited resolution of the data.

The final model for FabG contained two protein chains spanning residues Met1–Val241 for either chain, with gaps at Gly85–Asp99, Gly138–Gly141 and Lys182–Leu189 in chain *A* and Thr87–Asp99 and Ile139–Gly141 in chain *B*. The two protomers are very similar and superimposed with an r.m.s.d. of 0.44 Å. 129 water molecules were located. In refining this structure the inclusion of TLS terms did not significantly improve the refinement; hence, no anisotropic displace­ment factors were deposited. The final structure was validated with *Coot* and *MolProbity* (Chen *et al.*, 2010 ▶) and deposited in the PDB with the identifier 3f9i.

## 3. Results and discussion

### 3.1. Overall structure

**Figure 2:**
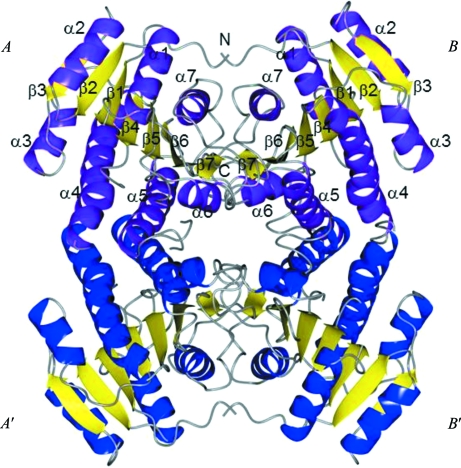
Crystal structure of *R. prowazekii* 3-ketoacyl-(ACP) reductase (FabG). FabG from *R. prowazekii* crystallizes with two molecules per asymmetric unit, which are shown as ribbon diagrams with purple helices and yellow strands. The active-site residues (Ser143, Tyr156 and Arg160) are grouped near the loop connecting α5 and β5. Secondary-structure elements are labeled from both chains. A tetramer is generated by crystallographic symmetry. Molecules *A*′ and *B*′ are shown as ribbon diagrams with blue helices and yellow strands. The figure was generated with *CCP*4*mg* (McNicholas *et al.*, 2011 ▶).

The structure of *R. prowazekii* FabG (Fig. 2 ▶) is very similar to those reported for *E. coli* FabG and other bacterial FabGs; the protomer assumes a Rossmann-fold structure with a seven β-­stranded twisted parallel β-sheet flanked by four α-helices on either side and two βαβαβ motifs at the core (Fig. 2 ▶). *R. prowazekii* FabG crystallizes with two protomers per asymmetric unit. A tetramer is generated by crystallographic symmetry. According to *PISA* analysis (Krissinel & Henrick, 1997 ▶) there are two types of interface: a larger interface exists between *AB* and *A*′*B*′ comprising 1690 Å^2^ with an estimated binding energy of −126 kJ mol^−1^ and a smaller interface exists between *A* *B*′ and *A*′*B* comprising 845 Å^2^ with an estimated binding energy of −71 kJ mol^−1^. For FabGs tetramers are typically observed. It is therefore assumed that *R. prowazekii* FabG is in a tetrameric state.

### 3.2. Comparison with other bacterial 3-ketoacyl-(ACP) reductases

**Figure 3:**
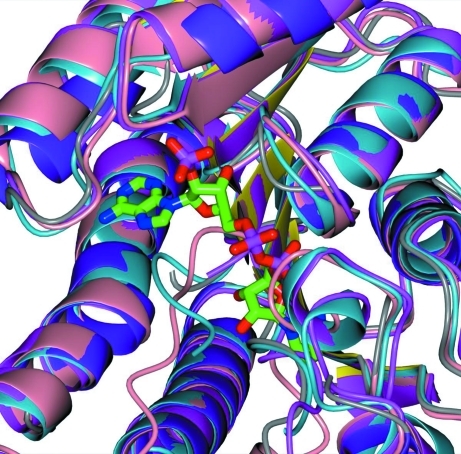
Structural superimpositions of monomeric units for FabG. In this figure, the monomers of FabG from *R. prowazekii* (PDB entry 3f9i; purple helices, yellow strands), from *E. coli* (PDB entry 1i01; magenta), from *M. tuberculosis* (PDB entry 1uzm; light blue) and the NADP^+^-bound structure from *V. cholerae* (PDB entry 3rsk, pink) are superimposed. There is a significant structural variability between these structures in the proximity of the NADP^+^-binding site.

**Figure 4:**
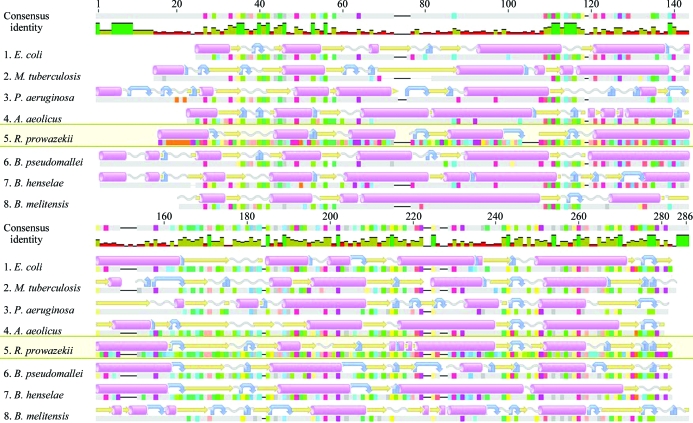
Alignment of solved structures of bacterial 3-ketoacyl-(ACP) reductases. Multiple sequence and secondary-structure alignment of bacterial orthologs. Numbering along the top is based on the alignment consensus. Predicted α-helices, β-strands, coils and turns are shown as pink cylinders, yellow arrows, grey corkscrews and blue curved arrows, respectively. Sequences are taken from the PDB. From top to bottom: *E. coli*, 1i01 (Price *et al.*, 2001 ▶); *M. tuberculosis*, 1uzm (Cohen-Gonsaud *et al.*, 2002 ▶); *Pseudomonas aeruginosa*, 2b4q (Miller *et al.*, 2006 ▶); *Aquifex aeolicus*, 2pnf (Q. Mao, R. Huether, W. L. Duax & T. C. Umland, unpublished work); *R. prowazekii*, 3f9i (this work); *Burkholderia pseudomallei*, 3ftp (Seattle Structural Genomics Center for Infectious Disease, unpublished work); *Bartonella henselae*, 3grp (B. L. Staker, unpublished work); *Brucella melitensis*, 3n74 (Seattle Structural Genomics Center for Infectious Disease, unpublished work). This figure was prepared with *Geneious* (Drummond *et al.*, 2010 ▶).

The *R. prowazekii* FabG shows 1.33 Å r.m.s.d. with the *E. coli* structure (PBD entry 1i01; Price *et al.*, 2001 ▶) over 205 residues, 1.26 Å r.m.s.d. with the *M. tuberculosis* structure (PDB entry 1uzm; Cohen-Gonsaud *et al.*, 2002 ▶) over 201 residues and 1.22 Å r.m.s.d. with the NADP^+^-bound *Vibrio cholerae* structure (PDB entry 3rsh; J. Hou, M. Chruszcz, D. Cooper, M. Grabowski, H. Zheng, T. Osinski, I. Shumilin, W. Anderson & W. Minor, unpublished work) over 208 residues, as shown in Fig. 3 ▶, whereas the r.m.s.d. with *Sus scrofa* mammalian FAS (PDB entry 2vz8; Maier *et al.*, 2008 ▶), used here as a homologue for the unsolved human FAS structure, is 2.5 Å over 80%. Although there are closer orthologs to *Rickettsia* FabG than the three aforementioned bacterial structures, these four structures represent prominent bacterial pathogens and the overall structural similarity is useful to note. Furthermore, a multiple sequence alignment (Fig. 4 ▶) of eight bacterial orthologs of 3-ketoacyl-(ACP) reductase ranges from 30 to 54% sequence identity over 93–95% sequence coverage. Of these eight orthologs, only one is not an NIAID Class A–C pathogen. Based on these high overall correspondences in sequence and secondary and tertiary structure, conclusions drawn from the other bacterial structures may also be applicable to the *Rickettsia* protein.

FabG has been shown to have negative cooperative binding of NADPH (Price *et al.*, 2001 ▶) and this effect is enhanced by the presence of acyl carrier protein (ACP). NADPH binding also increases the affinity and decreases the maximum binding of ACP to FabG. Thus, unlike other members of the short-chain dehydrogenase/reductase superfamily, FabG undergoes a substantial conformational change upon cofactor binding that organizes the active-site triad (Ser143, Tyr156 and Arg160) and alters the affinity of the other substrate-binding sites in the tetrameric enzyme (Price *et al.*, 2001 ▶). This behavior could explain why the pocket around the NADP^+^ in Fig. 3 ▶ differs significantly among the four bacterial targets and serves as a caveat that a given bacterial FabG antidote may not immediately be effective against *Rickettsia*.

While a number of antimicrobial options have been identified for targeting most of the enzymes in the FAS II pathway, FabG is still relatively unaddressed, in part owing to the conformational change that it undergoes. Epigallocatechin gallate (EGCG), along with other related plant polyphenols, has been shown to be a mixed-type inhibitor of *E. coli* FabG, although inhibiting fatty-acid synthesis was not the only target of EGCG (Zhang & Rock, 2004 ▶). Similar findings have been generated against the eukaryotic *Plasmodium falciparum* ortholog (Tasdemir *et al.*, 2006 ▶). Additionally, plant polyphenols have not been considered to be effective against Gram-negative bacteria owing to their effective permeability barrier as well as the action of efflux pumps; deactivating these pumps in conjunction with plant polyphenol administration has been shown to be effective (Tegos *et al.*, 2002 ▶). Nonetheless, Zhang and coworkers concluded that the flexible substrate-binding pocket, while a challenge to model for druggability (Price *et al.*, 2004 ▶), offers the prospect of utilizing a more potent slow-binding inhibitor (Zhang *et al.*, 2006 ▶). In contrast, Poncet-Montange and coworkers inferred from an inactivated mutant form of the *M. tuberculosis* FabG1 that drug-target screening could be among ligands that freeze the enzyme in its inactive form (Poncet-Montange *et al.*, 2007 ▶).

## 4. Conclusion

The SSGCID has solved a 2.25 Å resolution structure of *R. prowazekii* FabG, a 3-ketoacyl-(acyl-carrier-protein) reductase, which corresponds well to the solved structures for other notable bacterial pathogens while differing from mammalian analogs. For *Rickettsia* in particular this is a significant target for drug development as the organism has a minimal proteome with very few essential biosynthetic pathways maintained. Owing to a significant conformational change during substrate binding, it has been difficult to identify suitable inhibitors for this essential and highly conserved enzyme.

## Supplementary Material

PDB reference: 3-ketoacyl-(acyl-carrier-protein) reductase, 3f9i

## Figures and Tables

**Table 1:** Data-collection statistics Values in parentheses are for the highest resolution shell.

Space group	*P*4_1_2_1_2
Unit-cell parameters (Å)	*a* = *b* = 74.16, *c* = 183.01
Wavelength (Å)	1.5418
Resolution range (Å)	50–2.25 (2.31–2.25)
No. of unique reflections	24572 (1687)
Multiplicity	5.9 (2.3)
Completeness (%)	97.9 (93.0)
*R*_merge_†	0.056 (0.375)
Mean *I*/σ(*I*)	20.7 (2.3)

† *R*_merge_ = 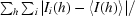 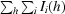.

**Table 2:** Refinement and model statistics

Resolution range (Å)	50–2.25
*R*_cryst_†	0.213
*R*_free_†	0.274
No. of reflections	24573
Completeness (%)	98.1
No. of reflections, test set	1256
Completeness, test set (%)	5.1
R.m.s.d. bonds (Å)	0.017
R.m.s.d. angles (°)	1.60
Protein atoms	3252
Nonprotein atoms	129
Mean *B* factor (Å^2^)	38.8
Residues in favored region	419 (96.3%)
Residues in allowed region	13 (3.0%)
Residues in disallowed region	3 (0.7%)
*MolProbity* score (percentile)	2.34 (67th)
PDB code	3f9i

† *R*_free_ = 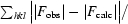 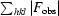. The free *R* factor was calculated using 5% of the reflections omitted from the refinement (Winn *et al.*, 2011 ▶).
